# The interplay of emotion expressions and strategy in promoting cooperation in the iterated prisoner’s dilemma

**DOI:** 10.1038/s41598-020-71919-6

**Published:** 2020-09-11

**Authors:** Celso M. de Melo, Kazunori Terada

**Affiliations:** 1 Computational and Information Sciences, U.S. Army Research Laboratory, 12015 Waterfront Drive, Building #3, Playa Vista, CA 90094-2536 USA; 2 Department of Electrical, Electronic and Computer Engineering, Gifu University, Gifu, Japan

**Keywords:** Psychology, Human behaviour

## Abstract

The iterated prisoner’s dilemma has been used to study human cooperation for decades. The recent discovery of extortion and generous strategies renewed interest on the role of strategy in shaping behavior in this dilemma. But what if players could perceive each other’s emotional expressions? Despite increasing evidence that emotion signals influence decision making, the effects of emotion in this dilemma have been mostly neglected. Here we show that emotion expressions moderate the effect of generous strategies, increasing or reducing cooperation according to the intention communicated by the signal; in contrast, expressions by extortionists had no effect on participants’ behavior, revealing a limitation of highly competitive strategies. We provide evidence that these effects are mediated mostly by inferences about other’s intentions made from strategy and emotion. These findings provide insight into the value, as well as the limits, of behavioral strategies and emotion signals for cooperation.

## Introduction

For many decades, the prisoner’s dilemma has been the main paradigm for the study of human cooperation^1–3^. Several strategies have been identified in this dilemma that influence cooperation^3–6^ including, more recently, extortion and generous “zero-determinant” strategies^7–11^. However, despite increasing evidence that emotion signals can influence decision making^12–14^, the effects of emotional expressions on behavior in the prisoner’s dilemma has received considerably less attention. Here we show that emotional expressions moderate the effect of generous strategies, increasing or reducing cooperation according to the intention communicated by the emotional signal. In contrast, emotion expressions by extortionists had no effect on participants’ behavior, revealing an important limitation of highly competitive strategies. Our results indicate that these effects are mostly mediated by participants’ expectations of cooperation made from the counterpart’s strategy and emotion, but also by the participants’ emotional experiences during the interaction. These findings provide insight into the importance, relative influence, as well as the limits, of behavioral strategies and emotion signals for emergence of cooperation. The results also have important practical applications for the design of increasingly pervasive autonomous machines—such as robots, self-driving cars, drones, and personal assistants—which will inevitably rely on cooperation with humans for their success^15–19^.

In the iterated prisoner’s dilemma, two players make, in each round, a simultaneous decision to either cooperate or defect. If they both cooperate, they each receive a payoff *R*. If they both defect, they receive a payoff *P* that is lower than *R*. However, if one cooperates and the other defects, the defector earns the highest possible reward (*T*) and the cooperator the lowest (*S*), i.e., *T* > *R* > *P* > *S*. If the number of rounds is finite, the rational prediction is that players should always defect^20^; however, in practice, people often cooperate^3,21^ and one of the main thrusts of research in the area has been finding strategies that can promote cooperation. Recently, Press and Dyson identified a class of strategies, so-called “zero-determinant,” that include strategies that unilaterally ensure a linear relation between one player’s payoff and the counterpart’s payoff^7^. On one extreme, there are extortion strategies^7,8,10^, which enforce that the counterpart cannot earn more than the extortionist by (a) cooperating less often than the counterpart, and (b) cooperating often enough that the most profitable response for the counterpart—albeit not as profitable as for the extortionist—is to cooperate. Extortion strategies, though, are only able to succeed under constrained settings^7,8^, tend to be evolutionary unstable^8,9^ and, in practice, are punished by humans^10^. On the other extreme, there are generous strategies, which reward cooperation while only punishing defection mildly^9^. Generous strategies are outperformed in head-to-head matches with extortion strategies but, tend to dominate in evolving heterogeneous populations^9^ and are rewarded, in practice, by humans^10,11^.

Whereas counterpart strategy can explain much variance in players’ behavior in the prisoner’s dilemma^3^, there is growing evidence that emotion expressions are very influential in shaping human decision making^12–14^. Since emotion signals tend to occur spontaneously, researchers have suggested they can be important in identifying cooperators^22, 23,24^. Expressions of emotions serve, in fact, important social functions, such as communicating one’s mental states and goals to others^25–28^. There is general agreement among emotion theorists that emotions are elicited by ongoing, conscious or nonconscious, appraisal of events with respect to the individual’s beliefs and goals^29–31^. Different emotions result from different appraisals, as well as their associated patterns of physiological manifestation, action tendencies, and behavioral expressions. Expressions of emotions, therefore, reflect differentiated information about the expresser’s appraisals and goals^12,13,32^. Accordingly, de Melo et al.^12^ showed that, in the iterated prisoner’s dilemma, participants successfully inferred from emotion expressions how counterparts’ were appraising the interaction and, from this information, made inferences about counterparts’ likelihood of future cooperation.

The effects of emotion expressions in extortion and generous strategies, however, have not been studied so far. When engaging with counterparts that follow a tit-for-tat strategy—i.e., only cooperate if the other cooperated in the previous round—de Melo and Terada^19^ showed that participants cooperated more or less according to whether the emotion expressions signaled a cooperative (e.g., joy following mutual cooperation) or competitive intention (e.g., joy following exploitation). Tit-for-tat is an interesting strategy as it strikes a balance between rewarding cooperation by the other player and punishing if the other player defects^4,5^. Given its inherently contingent nature, it is perhaps unsurprising that emotions expressions, being an important source of information about others’ mental states^12^, have a strong moderating effect. It is not clear, though, if similar patterns will occur with highly competitive strategies (e.g., extortion) or highly cooperative strategies (e.g., generous). On the one hand, when the emotion is incongruent (e.g., cooperative emotion displays with extortion behavior), people may be more motivated to process the information being communicated by emotion^13,33^, which would lead to a strong effect of emotion. On the other hand, people may simply interpret incongruent emotion displays as not being genuine and dismiss them^34^, which would lead to no effect of emotion. Here, thus, we study the moderating effects of emotion expressions in generous and extortion strategies.

**Figure 1:**
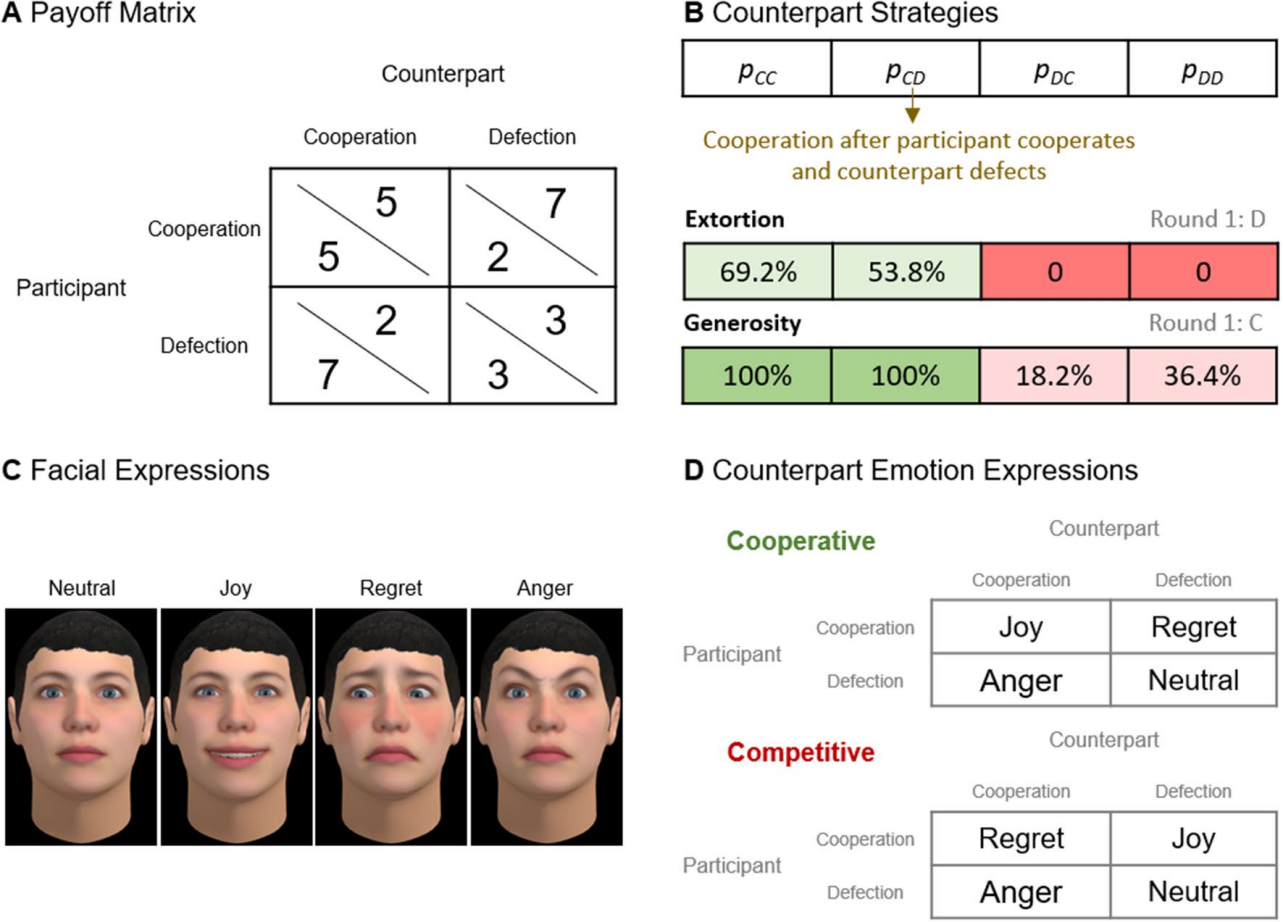
Experimental task and conditions. (**A**) The payoff matrix for the prisoner’s dilemma. Participants engaged in 20 rounds of this task. (**B**) Counterpart strategies are defined by the probabilities of cooperation following a specific outcome^10^. We consider the extortion (starting with defection) and generosity (starting with cooperation) strategies. (**C**) The validated facial expressions for the counterpart’s virtual representation in the task. (**D**) Two emotion expression patterns were considered: cooperative (e.g., joy following mutual cooperation) and competitive (e.g., joy following participant exploitation).

We present an experiment where participants engaged in the iterated prisoner’s dilemma with counterparts that followed extortion, and generosity strategies and showed cooperative and competitive emotion expressions. The payoff matrix we used, shown in Fig. 1A, has the following parameters: *T* = 7, *R* = 5, *P* = 3, and *S* = 2. To avoid any reputation effects, the experiment was fully anonymous—i.e., the participants were anonymous to each other and to the experimenters (please see the “Methods” section for details on how this was accomplished). Participants engaged in 20 rounds of the dilemma and were instructed that their final payoff was the sum of the points earned across all rounds. The points had real financial consequences as they would be converted to tickets for a $30 lottery (see “Methods” for details). Prior to starting the task, the participants were quizzed on these instructions and had to answer all questions correctly before proceeding.

Building on prior work^7–10^, the counterpart strategies were specified based on the probability of cooperation following each possible outcome of the prisoner’s dilemma; specifically, we followed the methodology of Hilbe et al.^10^ to define the probabilities shown in Fig. 1B. Please see the Supplemental Information (SI) for details and proof that the proposed strategies meet the requirements for zero-determinant strategies. The extortion strategy only cooperated with a 69.2% chance following mutual cooperation and 53.8% chance after exploiting the participant; otherwise, it would defect (including in the first round). The generosity strategy cooperated in the first round and when the counterpart cooperated in the previous round; moreover, it would still cooperate with a 18.2% chance after being exploited by the participant and 36.4% chance following mutual defection. Participants were instructed they would engage in the task with other participants but, to increase experimental control and implement these strategies precisely, participants engaged with a computer script. Similar methods have been used in previous research^15,19^, all experimental procedures were approved by the Gifu University IRB, and participants were fully debriefed at the end.

To support emotion expression, players were represented by virtual faces. Please see the SI for further details on the ecological validity of using virtual faces for this research and a brief overview of similar work using this methodology. The counterparts’ face always corresponded to a young white Caucasian character and, as shown in Fig. 1C, the facial displays showed prototypical expressions for joy, regret, and anger^12,31^—for a validation of the expressions with an independent participant sample and review of prior validation studies for similar expressions, please see the SI. The character and expressions were animated in real-time (please see the SI for a video S1 of the experimental software). Counterparts expressed emotion according to a cooperative and competitive orientation^12^, Fig. 1D: cooperative—joy following mutual cooperation, regret after exploiting the participant, anger after being exploited, and neutral otherwise; and, competitive—regret following mutual cooperation (given that it missed the opportunity to exploit the participant), joy after exploiting the participant, anger after being exploited and, neutral otherwise. After the round outcome was revealed but before seeing the counterpart’s emotional reaction, participants were asked “How do you feel about this outcome?” and were able to self-report which emotion they felt among joy, sadness, anger, regret, or neutral. The question, thus, was meant to encourage truthful reporting of experienced emotion (but see below for a question and results on whether participants believed the counterpart’s expressed emotion was genuine). Participants were instructed that they would be able to see the expressions from counterparts and vice-versa. To get insight on the inferences participants were making about the counterpart’s intentions, before the next round started, participants were asked how likely they thought the counterpart was to cooperate in the next round. Finally, after completing the task, to get further insight on whether participants were processing the emotional information, we asked, on a 1 (“Not at all”) to 7 (“Very much”) scale: “How mentally demanding was the task?”; and, “Were your counterpart’s emotions genuine?”.

## Results

**Figure 2:**
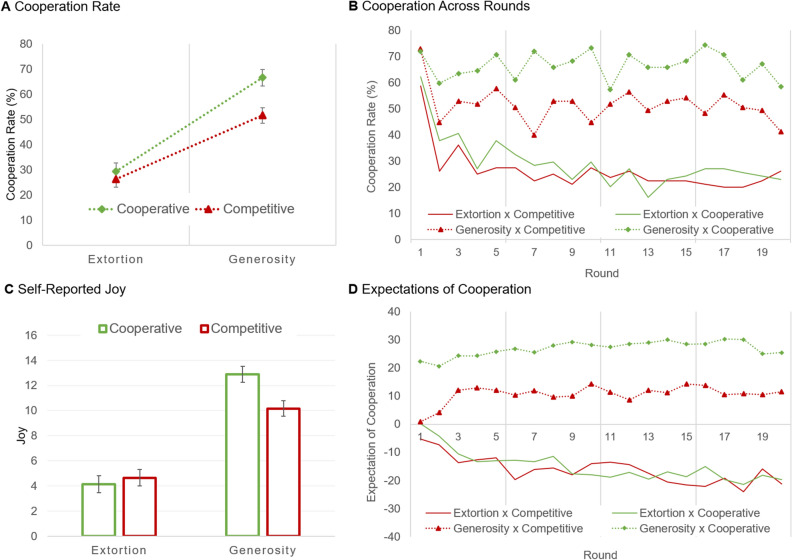
Participants’ cooperation rates, self-reports of joy, and expectations of cooperation in the iterated prisoner’s dilemma. (**A**) Cooperation rate was influenced by emotion expressions with the generosity strategy, but not with the extortion strategy. Error bars show standard errors. (**B**) Cooperation across rounds. (**C**) Participants reported the most joy with the generosity strategy and the least joy with the extortion strategy. Error bars show standard errors. (**D**) Expectations of cooperation for each condition.

The experiment, thus, followed a 2 × 2 between-participants factorial design: strategy (extortion vs. generosity) × emotion (cooperative vs. competitive). We recruited 321 participants from an online pool (see the “Methods” section for details about recruitment, sample size, and sample demographics). Our analysis focused on cooperation rate across all rounds, which is shown in Fig. 2A. A strategy × emotion ANOVA revealed a large effect of strategy, *F*(1, 317) = 92.06, *P* < 0.001, partial η^2^ = 0.225, and Bonferroni post-hoc tests showed that participants cooperated more with generosity than extortion. There was an effect of emotion, *F*(1, 317) = 7.70, *P* = 0.006, partial η^2^ = 0.024, and Bonferroni post-hoc tests confirmed that participants cooperated more with (expressively) cooperative than competitive counterparts. The results, therefore, reveal that strategy and emotion influenced the participants’ decisions and, moreover, that the effect of strategy was stronger than emotion.

There was also a trend for a strategy × emotion interaction, *F*(1, 317) = 3.35, *P* = 0.068, partial η^2^ = 0.010. Moreover, if instead of the conventional interaction test reported by the ANOVA, we run a planned synergistic contrast test for this interaction^35^, which predicts that the increase in cooperation occurs mostly for cooperative displays with the generosity strategy, we achieve a statistically significant interaction, *t*(119) = 7.51, *p* < 0.001; please see the “Methods” for further details on planned contrast tests. This result, thus, suggests that the influence of emotion varied according to strategy. To gather more insight, we split the data by strategy and ran independent samples *t* tests: for generosity, there was an effect of emotion, *t*(163) = 2.99, *P* = 0.003, *r* = 0.228; however, for extortion, there was no effect of emotion, *t*(152) = 0.76, *P* = 0.447. The results, thus, suggest that emotion signals influenced cooperation with generosity, but had no effect with extortion. To further understand the interaction, we ran factorial ANOVAs on the questions posed at the end of the task on experienced cognitive demand and whether the emotion expressions were perceived to be genuine. Regarding the former, there was a trend for an effect of strategy (*p* = 0.054), with increased cognitive demand for generosity (*M* = 3.50, *SE* = 0.16) than extortion (*M* = 3.05, *SE* = 0.17), and no effect of emotion (*p* = 0.798). Regarding the latter, there was an effect of strategy (*p* < 0.001), with participants perceiving emotions to be more genuine with generosity (*M* = 4.94, *SE* = 0.14) than extortion (*M* = 3.98, *SE* = 0.15), and no effect of emotion (*p* = 0.628). There was also a strategy × emotion interaction (*p* < 0.001), with participants perceiving competitive expressions to be equally genuine, but the cooperative displays to be more genuine for generosity than extortion. Altogether, these results suggest participants were actively processing the information from counterparts’ actions and emotions, but perceived emotions to be less genuine with extortion than generosity.

To get insight into how cooperation changed across rounds (Fig. 2B), we ran a round × strategy × emotion mixed ANOVA. The results indicated a main effect of round (*F*(16.21, 5,138.07) = 8.75, *P* < 0.001, partial η^2^ = 0.027), with cooperation starting high in the first round, then stabilizing lower until the last round, when it lowered further. There was also a round × strategy interaction (*F*(16.21, 5,138.07) = 3.11, *P* < 0.001, partial η^2^ = 0.010), with cooperation lowering much quicker with extortion than generosity. However, there were no statistically significant interactions involving round and emotion, thus suggesting that the effect of emotion was not significantly different across rounds.

To understand the mechanism driving the effects of strategy and emotion on cooperation rate, we build on prior work suggesting that the social effects of emotion can occur through inferential and affective processes^12,13^; in our case, we looked at participants’ self-reported emotion and expectations of cooperation. Regarding the former, we focused on self-reports of joy, as shown in Fig. 2C (but see the SI for a full analysis of all self-reported emotions). A factorial ANOVA revealed an effect of strategy, *F*(1, 317) = 122.45, *P* < 0.001, partial η^2^ = 0.279, with participants experiencing more joy with generosity than extortion. There was also a trend for an effect of emotion, *F*(1, 317) = 2.94, *P* = 0.087, partial η^2^ = 0.009, with participants tending to experience more joy with (expressively) cooperative than competitive counterparts. Regarding expectations of cooperation (Fig. 2D), a factorial ANOVA confirmed an effect of strategy, *F*(1, 317) = 150.43, *P* < 0.001, partial η^2^ = 0.322, with participants expecting more cooperation with generosity than extortion. There was also an effect of emotion, *F*(1, 317) = 9.79, *P* = 0.002, partial η^2^ = 0.030, with participants expecting more cooperation from (expressively) cooperative than competitive counterparts. These results, thus, indicate that participants made appropriate inferences about expectations of cooperation from strategy and emotion, while experiencing concomitant emotion in the process.

But, did expectations of cooperation and experienced emotion explain the participants’ decisions? To further understand this, we ran multiple mediation analyses on the effects of strategy and emotion on cooperation. A multiple mediation analysis^36^ is a statistical technique that helps establish causality by determining if certain mediators (e.g., expectations of cooperation) account for the effect of an independent variable (e.g., strategy) on a dependent variable (e.g., cooperation rate). Regarding strategy, this analysis revealed that the effect of strategy was mediated by expectations of cooperation (indirect effect: 0.205, *P* < 0.001) and experienced joy (indirect effect: 0.117, *P* < 0.001), with the total effect (0.312, *P* < 0.001) becoming statistically non-significant once the effect of the mediators was accounted for (direct effect: − 0.014, *P* = 0.641). Regarding emotion, the analysis showed that the effect of emotion was mediated by expectations of cooperation (indirect effect: 0.055, *P* = 0.007); the total effect (0.096, *P* = 0.010) became non-significant given the mediator (direct effect: 0.020, *P* = 0.397). Please see the “Methods” section for further details on this methodology; Figure S1 and Table S1 in the SI also show, respectively, the mediation models and bootstrapping confidence intervals. In sum, the evidence indicates that the effects of strategy and emotion on cooperation were mediated by expectations of cooperation and, to a lesser degree, experiences of joy.

## Discussion

The ability to infer intentions and predict the behavior of others is critical for the emergence of cooperation among strangers^24,37^. Whereas much prior work has focused on understanding what actions individuals should take when engaged in an iterated prisoner’s dilemma^4–11^, here we show that people will readily seek and use additional sources of information to identify cooperators, in particular, emotion expressions. Given that this nonverbal signal is pervasive in nature^29–31^, it is important to shed light on how strategy and emotion expressions interact with each other to promote cooperation, as we do here. Consistent with research indicating that emotion expressions serve important social functions^25–28^ and influence others’ decision making^12–14^, our results report a moderating effect of emotions on a zero-determinant generous strategy, similarly to what had been shown for tit-for-tat^12,19^. In contrast, with a zero-determinant extortion strategy, emotion signals had no effect, thus, revealing a limitation for extortionists; in this case, given the highly competitive nature of the strategy, participants appear to be reluctant to believe the emotional expressions of extortionists were genuine. This is in line with prior research indicating that inauthentic displays of emotion do not encourage cooperation^34^. These findings are also consistent with prior research indicating that the effects of emotion expressions are likely to occur in ambiguous circumstances^13,38^; by contrast, this effect is muted in situations where there is less uncertainty about others’ behavior, as is the case with extortionists.

Our findings suggest that behavior in the iterated prisoner’s dilemmas can be explained by inferences participants made, from strategy and emotion expressions, about the counterparts’ intentions. This is compatible with prior research indicating that people retrieve, from emotion expressions, pertinent information about others’ mental states and those inferences shape their decisions^12,13^. The results emphasize the contextual meaning of the emotion signal, as the same expression led to opposite effects on cooperation depending on the context in which it was shown (e.g., joy following mutual cooperation versus following participant exploitation). This reinforces that it is not the display per se that matters, but the information they communicate about others’ intentions^12,39^. However, our findings also showed that participants’ emotion mediated their decisions, albeit to a lesser degree. This is in line with research indicating that others’ emotions can, depending on the situation, lead to the experience of empathic or complementary emotions^13,40^, which in turn can influence decision making^14^.

The findings presented here provide insight into the interplay of actions and emotion in shaping human behavior and this has important practical implications. Autonomous machines that act on people’s behalf are poised to become pervasive in society^15–19^ but, for these machines to succeed and be adopted it is essential that people are able to trust and cooperate with them. Whereas simulating appropriate strategies in these machines is the natural starting point, here we emphasize that designers cannot afford to ignore nonverbal communication, in particular, emotion expressions^19,41,42^. Emotionally expressive machines can, additionally, be invaluable tools for the systematic study of human decision making, the influence of nonverbal signals, and the underlying psychological mechanisms, as demonstrated in our experiment. Finally, given that autonomous machines can be constructed to perform optimal actions and emotional expressions, they introduce a unique opportunity to help build a more cooperative society.

## Methods

This section describes details for the experimental methods that are not described in the main body of the text.

### Prisoner’s dilemma task

Similarly to previous work^12^, the prisoner’s dilemma task was recast as an investment game and described as follows to the participants: “You are going to play a two-player investment game. You can invest in one of two projects: project green and project blue. However, how many points you get is contingent on which project the other player invests in. So, if you both invest in project green, then each gets 5 points. If you choose project green but the other player chooses project blue, then you get 2 and the other player gets 7 points. If, on the other hand, you choose project blue and the other player chooses project green, then you get 7 and the other player gets 2 points. A fourth possibility is that you both choose project blue, in which case both get 3 points”. Thus, choosing project green corresponded to cooperation, and project blue to defection. A video of the software is available in the SI.

### Participant sample

Participants were recruited from an online pool—Amazon Mechanical Turk. Previous research shows that studies performed in online platforms can yield high-quality data and successfully replicate the results of behavioral studies performed in traditional pools^43^. To estimate sample size, we followed the power calculations proposed by Cohen^44^ and implemented in G*Power^45^—a software that is often used by behavioral researchers. Based on earlier work^12,19^, we predicted a small to medium effect size (Cohen’s *f* = 0.20). Thus, for α = 0.05 and statistical power of 0.95, the recommended total sample size was 327 participants. We aimed to recruit 340 participants (85 per condition) but, as is common when running experiments in this pool, there were some participants that did not successfully complete the task or otherwise made data entry errors. In practice, we had to exclude 19 participants and we ended with a valid set of 321 participants: extortion × cooperative, *n* = 74; extortion × competitive, *n* = 80; generosity × cooperative, *n* = 82; generosity × competitive, *n* = 85). All participants were recruited from the United States and had an approval rate, based on prior work in the online pool, of at least 95%. The demographics distribution was as follows: gender—61.4% males; age distribution—18 to 21 years, 1.6%; 22–34 years, 51.4%; 35–44 years, 24.6%; 45–54 years, 14.3%; 55–64 years, 6.2%; over 64 years, 1.9%; ethnicity distribution—Caucasian, 71.3%; African American, 18.4%; East Indian, 0.6%; Hispanic or Latino, 7.5%; Southeast Asian, 2.2%.

### Financial incentives

Participants were paid for participation ($2.50), but also had the chance to earn extra money based on their performance. Accordingly, the total amount of points earned in the task, summed across all rounds, was converted to lottery tickets for a $30.00 lottery. After all participants in our sample completed the experiment, one lottery ticket was selected from the entire pot, representing a single participant.

### Full anonymity

Preserving full anonymity is important to minimize any reputation effects, such as participants’ concern for retaliation due to the decisions in the experiment. To accomplish full anonymity, first, counterparts were referred to by anonymous names (e.g., “Anonymous43”) and we also did not collect other information that would allow participant identification. Second, the experiment was anonymous to experimenters in that the online pool preserves participant anonymity unless the experimenters explicitly ask for identifying information from participants, which we did not.

### Data analyses

As reported in the main text, to study the effect of strategy and emotion on cooperation, experienced emotions, and expectations of cooperation, we ran strategy × emotion ANOVAs on the respective dependent variables. To understand the dynamics of cooperation across rounds, we ran a round × strategy × emotion mixed ANOVA with a Huynh–Feldt correction to account for a violation of the sphericity assumption. To understand effect size for any main effect or interaction in the ANOVA analyses, we report corresponding partial η^2^ values (following Cohen’s recommendations: 0.01, small; 0.09, medium; 0.25, large). Post-hoc tests were adjusted with Bonferroni corrections. Regarding the interaction for cooperation, the conventional analysis for our 2 × 2 ANOVA tests if the means for generosity and extortion strategies cross each other at different levels of the emotion factor; this results in a *P* value of 0.068. However, based on our theoretical motivation, a synergistic interaction^35^ would be more appropriate as it tests if the mean for generosity × cooperative is higher than for any of the other combination of the factors. This triangular pattern is a better theoretical fit than a crossing pattern. Accordingly, when we run this planned contrast for the interaction, we get a *P* value that is less than 0.001. Independent *t* tests were used to study the impact of emotion per strategy. To understand the effect size for these analyses, we report the Pearson’s correlation coefficient *r* (following Cohen’s recommendation: 0.10, small; 0.30, medium; 0.50, large).

For the multiple mediation analyses we ran binary comparisons for strategy (extortion vs. generosity) and emotion (competitive vs. cooperative); the first level was coded as 1, and the second level as 0. The mediators were expectations of cooperation and self-reported experiences of joy, sadness, regret, and anger. The dependent variable was cooperation rate. To determine mediation, we focused on the 95% bootstrapping confidence intervals; when the interval did not include zero, it can be argued that the respective mediator played a role in mediating the corresponding effect^36^.

### Human-subjects protection

All experimental methods were approved by the Medical Review Board of Gifu University Graduate School of Medicine (IRB ID#2018-159). As recommended by the IRB, written informed consent was provided by choosing one of two options in the online form: (1) “I am indicating that I have read the information in the instructions for participating in this research and have had a chance to ask any questions I have about the study. I consent to participate in this research.”, or (2) “I do not consent to participate in this research.” All participants gave informed consent and, at the end, were debriefed about the experimental procedures. All the experiment protocols involving human subjects was in accordance to guidelines of the Declaration of Helsinki.

## Supplementary information

Supplementary Appendix S1.

Supplementary Data S1.

Supplementary Figure S1.

Supplementary Figure S2.

Supplementary Video S1.

Supplementary Table S1.

## Data Availability

The authors declare that data supporting the findings of this study is available with the “Supplementary materials”.
