# Supplementary material for: The interplay of emotion expressions and strategy in promoting cooperation in the iterated prisoner’s dilemma
Source: Sci Rep. 2020 Sep 11;10:14959. doi: 10.1038/s41598-020-71919-6 (PMC7486426; doi:10.1038/s41598-020-71919-6)
Supplement: Supplementary file 1 — Supplementary Appendix S1. [file 41598_2020_71919_MOESM1_ESM.docx]

**Supplemental Information**

The Interplay of Emotion Expressions and Strategy in Promoting Cooperation in the Iterated Prisoner’s Dilemma

Celso M. de Melo, Kazunori Terada

# Generosity and Extortion Zero-Determinant Strategies

Zero-Determinant (ZD) strategies are memory-one strategies in which the decision for the current round only depends on the outcome of the previous round and they enforce a linear relationship between the players’ payoffs in the prisoner’s dilemma (*7*). ZD strategies are written as a 5-tuple ${(p}_{0},p_{R},p_{S},p_{T},p_{P})$, where $p_{0}$ is the player’s probability of cooperation in the first round ($m=1$), $p_{i}$ is the probability of cooperation in round $m\geq2$ given the payoff $i\in\left\{ R,S,T,P \right\}$ in the previous round. Payoff $R$ and $S$ are given to both players when both player cooperate and defect, respectively. If one player cooperates and the other defects, $T$ is given to the defector and $S$ is given to the cooperator. The relation $T>R>P>S$ is typically assumed to hold. According to Hilbe et al. (*10*), the probabilities of cooperation are defined as follows:

| $p_{R}=1-\phi\left( 1-s \right)\left( R-l \right)$  $p_{S}=1-\phi\left[ \left( 1-s \right)\left( S-l \right)+T-S \right]$  $p_{T}=\phi\left[ \left( 1-s \right)\left( l-T \right)+T-S \right]$  $p_{P}=\phi\left( 1-s \right)\left( l-P \right)$ | *(1)*  *(2)*  *(3)*  *(4)* |
| --- | --- |

, where $l$, $s$, and $\phi$ are constants.

While ZD strategies are able to enforce a linear relationship between average payoﬀ $\pi$ of the ZD strategist and the expected payoff $\tilde{\pi}$ of the counterpart when the game is repeatedly and infinitely played, Hilbe et al. (*10*) showed that when the game is played $M$ rounds, the relationship between $\pi$ and $\tilde{\pi}$ follows these inequalities:

| $-\frac{p_{0}}{\phi M}\leq\left( 1-s \right)l+s\pi-\tilde{\pi}\leq\frac{1-p_{0}}{\phi M}$ | *(5)* |
| --- | --- |

.

We used the payoff values $T=7$, $R=5$, $P=3$, $S=2$, and a total number of rounds $M=20$. The following are the values in our experiment for the constants in Equations (1)-(4), and the relation between $\pi$ and $\tilde{\pi}$ predicted by the inequalities in (5):

Extortion

$l=P$, $s=\frac{1}{3}$, $\phi=\frac{3}{13}$

$$p_{0}=0.000, p_{R}=0.692,p_{S}=0.000,p_{T}=0.538,p_{P}=0.000$$

$$\frac{1}{3}\cdot\pi+\frac{2}{3}\cdot3-\frac{13}{60}\leq\tilde{\pi}\leq\frac{1}{2}\cdot\pi+\frac{2}{3}\cdot3$$

Generosity

$l=R$, $s=\frac{1}{3}$, $\phi=\frac{3}{11}$

$$p_{0}=1.000, p_{R}=1.000,p_{S}=0.182,p_{T}=1.000,p_{P}=0.364$$

$$\frac{1}{3}\cdot\pi+\frac{2}{3}\cdot5\leq\tilde{\pi}\leq\frac{1}{2}\cdot\pi+\frac{2}{3}\cdot5+\frac{11}{60}$$

We conducted computer simulations to confirm that the strategies used in our experiment met the zero-determinant requirements. Figure S2-A shows that average payoﬀs $\pi$ and $\tilde{\pi}$ are distributed within the range of the linear relationship given by the inequalities in (5). Figure S2-B shows the comparison of experimental results to theoretical predictions, confirming that the relationship between the payoffs of the ZD strategist and the participants ﬁts the linear relationship prediction.

# Ecological Validity of Virtual Faces Stimuli

From the early 2000s, researchers have noted the potential of virtual technology as a methodological tool for the behavioral sciences^[[1]](#footnote-23061)^. This technology introduces several advantages: it allows high experimental control, it is high in mundane realism, and is cheaper than traditional methods (e.g., human confederates). Building on this work, de Melo, Carnevale, Read, and Gratch (*12*) proposed using virtual faces to study the impact of emotion expressions on human decision making. Compared to open-ended face-to-face interaction among participants, this method allows higher experimental control – as it is possible to systematically study the effect of precise expressions. Compared to using human confederates – i.e., experimenters that ostensibly engage in the experiment as other participants but follow scripted behavior – this method is cheaper and avoids the introduction of inadvertent noise introduced by confederates due to subtle changes in verbal and nonverbal behavior. Other pragmatic advantages include facilitating replication of the experimental manipulation and access to broader samples as it can easily run online. Several experiments have now been conducted using virtual faces to study human decision making – please see (*12*) and (*19*) for examples, as well as some of the referred work for additional examples.

# Validation of Virtual Face Expressions

We conducted an experiment to validate if the virtual faces’ emotion expressions were being perceived as expected. To accomplish this, we recruited a sample of 100 participants using an online pool (Amazon Mechanical Turk). The demographics for these samples were similar to the main experiment. Participants were shown pictures of the expressions for neutral, joy, regret, and anger in the Caucasian face. For each picture, they were asked to choose which emotion was best represented from five options: joy, regret, sadness, anger, and neutral. The expressions for neutral, joy, and anger were mostly perceived as the intended emotion: neutral, *χ^2^*(3) = 126.72, *P* < 0.001, 73% true positive rate; joy, *χ^2^*(3) = 246.64, *P* < .001, 93% true positive rate; and, anger, *χ^2^*(3) = 261.44, *P* < 0.001, 95% true positive rate. The expression of regret was equally recognized as regret (48%) as sadness (44%), *χ^2^*(3) = 71.60, *P* < .001; however, prior work (*12*) indicates that the effects of facial and verbal expressions of sadness and regret on cooperation rate in the prisoner’s dilemma are similar.

In addition to this validation sample, we additional validation of similar facial expressions is reported in our prior work (*12*, *19*). One validation sample looked at perception of emotion in similar expressions for Caucasian and Japanese faces using forced-choice questions (*19*). Complementary, we ran an additional validation study using 7-point Likert scale questions for perceptions of emotions (*12*). In general, the results of these prior studies are consistent with the validation study conducted for this work.

# Extended Analysis for Participants’ Self-Reported Emotions

In the main text, we presented an analysis of participants’ self-reported joy indicating that participants experienced more joy with the generosity strategy than with extortionists; moreover, participants tended to report more joy with expressively cooperative than competitive counterparts. Here we extend this analysis with additional strategy × emotion ANOVAs for self-reports of sadness, anger, regret, and neutral emotion. We only report the significant effects. Regarding sadness, there was a main effect of strategy, *F*(1, 317) = 4.96, *P* = 0.027, partial η^2^ = 0.015, with participants expressing less sadness with generosity than extortion. Regarding anger, there was a main effect of strategy, *F*(1, 317) = 4.63, *P* = 0.032, partial η^2^ = 0.014, with participants expressing more anger with extortion than generosity. Regarding regret, there were no statistically significant effects. Finally, regarding neutral emotion, there was a main effect of strategy, *F*(1, 317) = 71.18, *P* < 0.001, partial η^2^ = 0.183, with participants expressing no emotion more often with extortion than generosity.

1. Blascovich, J., Loomis, J., Beall, A., Swinth, K., Hoyt, C. & Bailenson, J. Immersive virtual environment technology as a methodological tool for social psychology. Psychol. Inq. **13**, 103-124 (2002). [↑](#footnote-ref-23061)
