# Supplementary material for: The interplay of emotion expressions and strategy in promoting cooperation in the iterated prisoner’s dilemma
Source: Sci Rep. 2020 Sep 11;10:14959. doi: 10.1038/s41598-020-71919-6 (PMC7486426; doi:10.1038/s41598-020-71919-6)
Supplement: Supplementary file 3 — Supplementary Figure S1. [file 41598_2020_71919_MOESM3_ESM.docx]

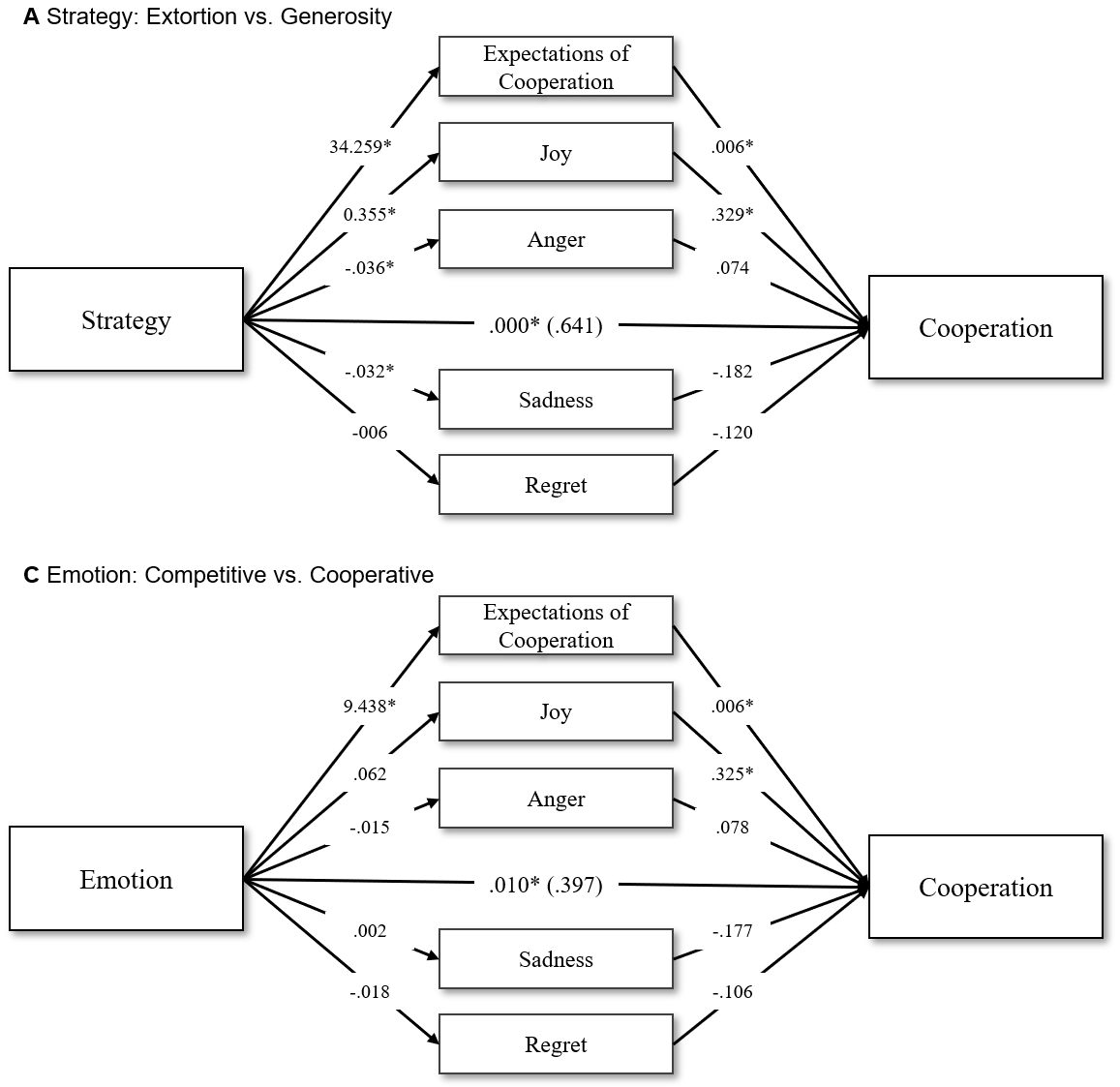


**Fig. S1.** Multiple mediation models for the effects of strategy and emotion on cooperation. Each model tests five possible mediators: expectations of cooperation and the participant’s self-reported joy, anger, sadness, and regret. The left arrows connecting the independent variable (IV) to mediators indicate the direct effect of the IV on the mediators (typically called *a* path). The right arrows connecting the mediators to the dependent variable (DV) indicate the direct effect of the mediator on the DV (typically called *b* path). The arrow connecting the IV to the DV indicates the total effect of the IV on the DV (typically called *c* path) and, in parenthesis, the direct effect of the IV on the DV (typically called *c’*). Multiple mediation occurs when the total effect is significant but the direct effect is not, suggesting that (some of) the mediators account for the effect (*36*). * *P* < 0.05
