# Supplementary material for: The interplay of emotion expressions and strategy in promoting cooperation in the iterated prisoner’s dilemma
Source: Sci Rep. 2020 Sep 11;10:14959. doi: 10.1038/s41598-020-71919-6 (PMC7486426; doi:10.1038/s41598-020-71919-6)
Supplement: Supplementary file 4 — Supplementary Figure S2. [file 41598_2020_71919_MOESM4_ESM.docx]

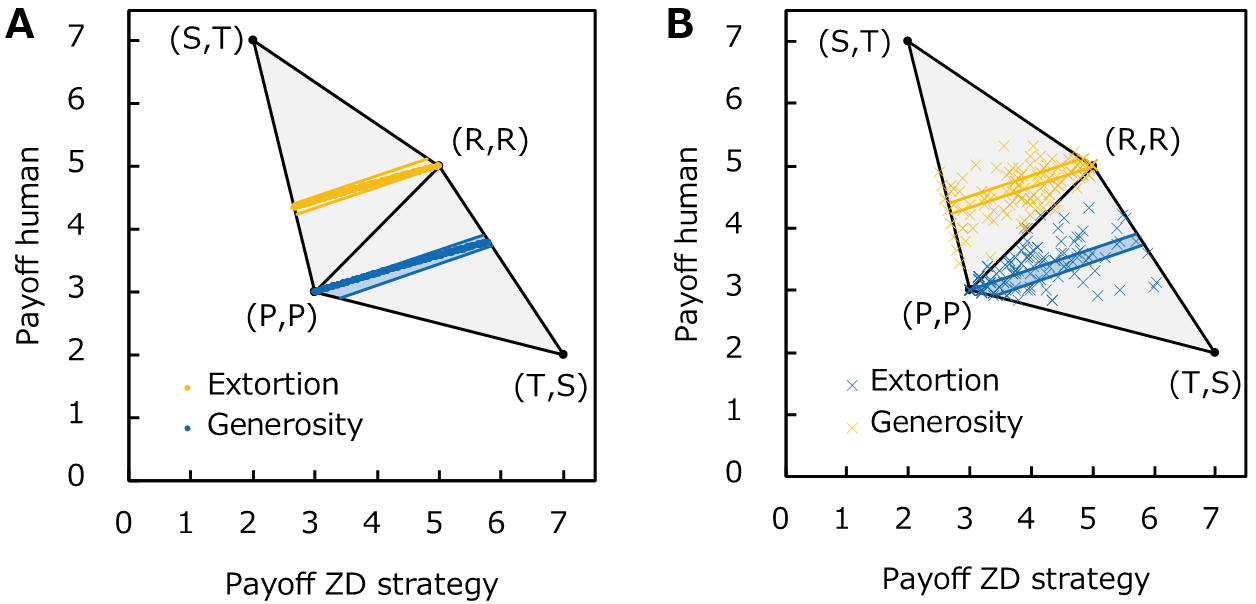


**Fig. S2.** Comparison of experimental results to theoretical predictions and simulations for the generosity and extortion strategies. A gray-shaded rectangular area surrounded by a solid line indicates the space of possible payoffs for the two players of the prisoner’s dilemma. X-axis and y-axis indicate payoffs of ZD strategy and counterpart, respectively. The color-shaded areas between two straight colored solid lines indicate expected payoff ranges according to the inequalities in (5) – i.e., the theoretical predictions. (**A**) Comparison of simulated payoffs to the theoretical prediction. Each dot between two color solid lines indicates average payoff of 1,000 simulations of the prisoner’s dilemma for a fixed cooperation rate (randomly chosen from 0 to 1). (**B**) Comparison of experimental results to theoretical predictions.
