# Supplementary material for: The interplay of emotion expressions and strategy in promoting cooperation in the iterated prisoner’s dilemma
Source: Sci Rep. 2020 Sep 11;10:14959. doi: 10.1038/s41598-020-71919-6 (PMC7486426; doi:10.1038/s41598-020-71919-6)
Supplement: Supplementary file 6 — Supplementary Table S1. [file 41598_2020_71919_MOESM6_ESM.docx]

Table S1. Bootstrapping Analysis of the Statistical Significance of the Indirect Effects.

| Indirect Effect | Point Estimate | Standard Error | 95% Confidence Interval | |
| --- | --- | --- | --- | --- |
|  |  |  | Lower Bound | Upper Bound |
| *Strategy (Extortion vs. Generosity)* | | | | |
| Total | .326 | .033 | .263 | .391 |
| Expectations of cooperation | .205 | .030 | .153 | .268 |
| Joy | .117 | .027 | .069 | .177 |
| Anger | .003 | .004 | -.012 | .002 |
| Sadness | .006 | .005 | .000 | .020 |
| Regret | .001 | .003 | -.002 | .009 |
| *Emotion (Competitive vs. Cooperative)* | | | | |
| Total | .076 | .030 | .018 | .132 |
| Expectations of cooperation | .055 | .021 | .016 | .098 |
| Joy | .020 | .014 | -.003 | .051 |
| Anger | -.001 | .002 | -.011 | .001 |
| Sadness | .000 | .003 | -.008 | .005 |
| Regret | .002 | .003 | -.003 | .011 |
